# Neural computations in prosopagnosia

**DOI:** 10.1093/cercor/bhae211

**Published:** 2024-05-24

**Authors:** Simon Faghel-Soubeyrand, Anne-Raphaelle Richoz, Delphine Waeber, Jessica Woodhams, Roberto Caldara, Frédéric Gosselin, Ian Charest

**Affiliations:** Département de psychologie, Université de Montréal, 90 av. Vincent D’indy, Montreal, H2V 2S9, Canada; Department of Experimental Psychology, University of Oxford, Anna Watts Building, Woodstock Rd, Oxford OX2 6GG; Département de psychologie, Université de Fribourg, RM 01 bu. C-3.117Rue P.A. de Faucigny 21700 Fribourg, Switzerland; Département de psychologie, Université de Fribourg, RM 01 bu. C-3.117Rue P.A. de Faucigny 21700 Fribourg, Switzerland; School of Psychology, University of Birmingham, Hills Building, Edgbaston Park Rd, Birmingham B15 2TT, UK; Département de psychologie, Université de Fribourg, RM 01 bu. C-3.117Rue P.A. de Faucigny 21700 Fribourg, Switzerland; Département de psychologie, Université de Montréal, 90 av. Vincent D’indy, Montreal, H2V 2S9, Canada; Département de psychologie, Université de Montréal, 90 av. Vincent D’indy, Montreal, H2V 2S9, Canada

**Keywords:** prosopagnosia, EEG, RSA, artificial neural networks, semantic representations

## Abstract

We report an investigation of the neural processes involved in the processing of faces and objects of brain-lesioned patient PS, a well-documented case of pure acquired prosopagnosia. We gathered a substantial dataset of high-density electrophysiological recordings from both PS and neurotypicals. Using representational similarity analysis, we produced time-resolved brain representations in a format that facilitates direct comparisons across time points, different individuals, and computational models. To understand how the lesions in PS’s ventral stream affect the temporal evolution of her brain representations, we computed the temporal generalization of her brain representations. We uncovered that PS’s early brain representations exhibit an unusual similarity to later representations, implying an excessive generalization of early visual patterns. To reveal the underlying computational deficits, we correlated PS’ brain representations with those of deep neural networks (DNN). We found that the computations underlying PS’ brain activity bore a closer resemblance to early layers of a visual DNN than those of controls. However, the brain representations in neurotypicals became more akin to those of the later layers of the model compared to PS. We confirmed PS’s deficits in high-level brain representations by demonstrating that her brain representations exhibited less similarity with those of a DNN of semantics.

## Introduction

The human brain is equipped with sophisticated machinery optimized to quickly and effectively recognize faces in a series of computations unfolding within tens of milliseconds. A dramatic contrast to this typically efficient process has been revealed in brain-lesioned patients with an inability to recognize faces, individuals called acquired prosopagnosics (Bodamer 1947). Findings from these patients have refined the functional role and the distributed nature of the face-sensitive brain regions in the ventral stream, such as the fusiform gyrus (fusiform face area [FFA]; Bobes et al. 2003; Kanwisher et al. 1997) and the lateral portion of the inferior occipital gyrus (Occipital Face Area, OFA; Dricot et al. 2008; Gauthier et al. 2000; Rossion et al. 2003; Sorger et al. 2007). This literature has generally contributed to the idea that specialized and category-selective neural modules are necessary for functional aspects of face processing (Cohen et al. 2019). Brain imaging findings from individuals born with deficits in face recognition (developmental prosopagnosics; (McConachie 1976; Avidan et al. 2005; Kaltwasser et al. 2014; Rosenthal et al. 2017; Jiahui et al. 2018) have revealed finer-grained functional neural differences in the processes associated with deficits in face recognition (Avidan et al. 2014; Rosenthal et al. 2017; Jiahui et al. 2018; Zhao et al. 2018). Overall, the cumulation of these neuropsychological, neuroanatomical, and functional components of prosopagnosia (Busigny et al. 2010; Dricot et al. 2008; B. C. Duchaine and Nakayama 2006; Rossion 2018; Rossion et al. 2003) has significantly contributed to neural models of face perception in the last two decades (B. Duchaine and Yovel 2015; Haxby et al. 2000; White and Mike Burton 2022). Yet, little is known on the nature of face representations of those patients (e.g. Caldara et al. 2005; Fiset et al. 2017), and next to nothing is known on the nature of brain dynamics and neural computations affected in prosopagnosia. Here, we report an investigation of the neural computations involved in the processing of faces and objects of patient PS, a well-documented case of pure acquired prosopagnosia (Rossion et al. 2003; Sorger et al. 2007), using Representational Similarity Analysis (RSA; Charest et al. 2014; Kriegeskorte, Mur, and Bandettini, 2008; Kriegeskorte and Kievit 2013; Nili et al. 2014) applied to brain imaging and computational models.

Patient PS is a right-handed woman who suffered a closed head injury in 1992, resulting in extensive bilateral occipitotemporal lesions that encompasses the right occipital face area (OFA), left FFA, and a small region of the right middle temporal gyrus (Sorger et al. 2007; Dricot et al. 2008). She stands out as one of the most extensively studied cases of acquired prosopagnosia, with over 32 scientific publications dedicated to her in the past two decades, as highlighted in recent reviews by Rossion (2022a, 2022b). The prominence of this case stems from the relatively focal nature of her lesions in the face network and the highly specific impairment she experiences in face identification (Busigny et al. 2010). Despite the comprehensive examination of her condition, including its impact on various perceptual mechanisms like holistic processes and the visual content of face representations (Caldara et al. 2005; Ramon et al. 2016; Fiset et al. 2017), there has been no direct attempt, to the best of our knowledge, to characterize the neural computations characteristic of her deficits. The assessment of the nature and extent of brain computations affected in prosopagnosia has traditionally relied on examining the temporal dynamics and face-selectivity of neural activity. For instance, event-related potential differences occurring later in processing have been viewed as indicative of higher-level processes than those occurring earlier (Eimer et al. 2012; Bentin and Deouell 2000; Herzmann et al. 2004; Gosling and Eimer 2011; Alonso Prieto et al. 2011; Simon et al. 2011; Tanaka et al. 2006; Liu-Shuang et al. 2016; Wiese et al. 2019).

Associations have been found between prosopagnosia and well-established neural markers of face processing, such as the face-sensitive N170 (Bentin et al. 1996) and face-selective fMRI activation (Towler and Eimer 2012; Bobes et al. 2003; Alonso Prieto et al. 2011; Gao et al. 2019). It is worth noting that despite her significant lesions and difficulties in face identification, PS still exhibits typical face-selectivity in spared regions of the right hemisphere, including the presence of a right FFA (Rossion et al. 2003; Gao et al. 2019), as well as a typical N170 component in the right hemisphere, but not in the left (Alonso-Prieto, 2011; Bobes et al. 2003; Dalrymple et al. 2011). Similarly, developmental prosopagnosics demonstrate typical activation in the core posterior regions of the face-processing system, including the OFA and FFA (Avidan et al. 2014). Recent advances in experimental techniques, such as fast periodic visual stimulation (Liu-Shuang et al. 2016b), have shed light on the crucial deficits in neural face individuation observed in PS. However, characterizing the underlying computations involved in these neural and perceptual processes remains a difficult endeavor. Describing neural computations is inherently challenging due to signal-to-noise ratio (SNR) issues, which are even more pronounced when recording brain activity from patients with brain lesions (Liu-Shuang et al. 2016a). Brain damage can substantially alter the flow of brain activity compared to typical observers, potentially distorting event-related potential components (Alonso Prieto et al. 2011) and requiring additional repetitions of conditions. Moreover, relying solely on temporal evidence has limitations as it only partially reveals the nature of the computations that the brain depends on (Lamme and Roelfsema, 2000; McDermott et al. 2002). Individuals performing different neural computations on faces may very well exhibit identical activity at a given latency, as indicated by univariate event-related potentials.

In recent years, innovative techniques have gained popularity for exploring the nature of brain representations by linking functional and multivariate brain activity with computational models (Dwivedi et al. 2021; di Oleggio Castello et al. 2021; Popham et al. 2021; Kriegeskorte and Diedrichsen 2016; Doerig et al. 2022; Faghel-Soubeyrand et al. 2024). The previously mentioned SNR concerns may explain why most studies on prosopagnosia have traditionally relied on a limited set of stimuli conditions, block-designs, and univariate methods such as averaging and subtraction. Although these methods are associated with relatively good statistical power, they have limited our understanding of the brain computations that underlie prosopagnosia. To gain a more comprehensive understanding, researchers have begun investigating brain processing using condition-rich designs (Allen et al. 2022; Charest et al. 2014a; Kriegeskorte and Kievit 2013; Naselaris et al. 2021). These approaches emphasize examining diverse models on a whole-brain basis, thus providing a broader description of the underlying brain mechanisms (Dwivedi et al. 2021; Kriegeskorte and Diedrichsen, 2019; Popham et al. 2021).

Here, we adopted a data-driven approach. We recorded the brain activity of patient PS and neurotypical controls in response to images from several categories. Using a well-established multivariate technique, RSA (Kriegeskorte, Mur, and Bandettini, 2008), we generated functional brain representations in a versatile format that enables direct comparisons across time, individuals with differing neuroanatomical structures, and computational models (Golarai et al. 2015; Popal et al. 2019). To understand how the lesions in PS’s ventral stream affect the temporal evolution of these brain representations, we conducted a comparison of the temporal generalization of these representational geometries in both PS and a control group. Additionally, to gain insights into the specific computational deficits in PS’s brain, we conducted a comparative analysis of her brain representations with those generated by artificial models designed to perform various types of computations. These models consisted in deep neural networks (DNN) specializing in vision and in semantics, providing a valuable perspective on the neural computations involved in PS’s deficits (see Faghel-Soubeyrand et al. 2024).

## Materials and methods

### Patient PS and neurotypical participants

A total of 20 participants were recruited for this study. The first group consisted of 19 neurotypicals individuals that included 15 young controls (9 female, M_age_ = 22.9 years old) as well as four age-matched controls (three female, M_age_ = 67.5). This sample size was chosen according to the effect sizes described in previous multivariate pattern analysis studies (Carlson et al. 2013; Cichy et al. 2014; Hebart et al. 2018; Faghel et al. 2022), as well as previous studies on prosopagnosia (Humphreys et al. 2007; Richoz et al. 2015; Liu-Shuang et al. 2016; Gao et al. 2019). Data from 10 of these young controls (#1–10) have been reported in a previous study (Faghel-Soubeyrand et al. 2024). One participant from the age-matched group (#2) was rejected due to faulty EEG recordings and poor behavioral performance during the one-back task and CFMT+. This study was approved by the Ethics and Research Committee of the University of Birmingham, The University of Fribourg, and informed consent was obtained from all participants.

### PS’s case report

Patient PS was born in 1950 and is a *pure* case of acquired prosopagnosia. She was hit by the side mirror of a London’s bus in 1992 while crossing the road. This closed head injury led to major lesions in the left middle fusiform gyrus, where the left Fusiform Face Area (lFFA) is typically located, and in the right inferior occipital gyrus, which typically locates the right Occipital Face Area (rOFA; see Gao et al. 2019) for converging fMRI evidence). Both regions play a critical functional role within the face cortical network (Rossion 2022a, 2022b). She also reported minor damages in the right middle temporal gyrus and left posterior cerebellum (for an exhaustive anatomical description and an illustration of her brain damages (see Sorger et al. 2007, Figs. 2 and 3). Patient PS is a very well-documented and described case of acquired prosopagnosia. She has been extensively studied over the last 20 years, leading to impactful scientific contributions that significantly enriched the theoretical models on human face perception (Rossion 2008, 2014; for a complete case report see, Rossion 2022a, 2022b; Rossion et al. 2003).

**Fig. 1:**
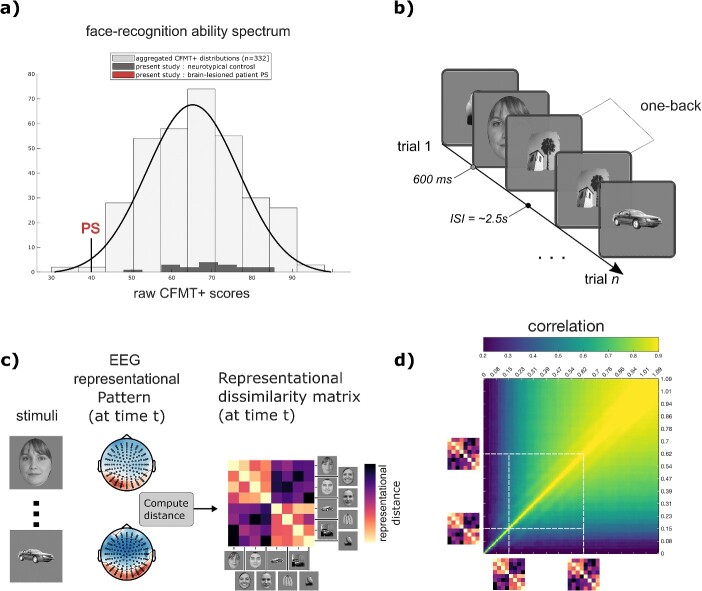
Overview of the experiment. **a)** the histogram shows the Cambridge face memory test long-form (CFMT+, (Russell et al. 2009) scores for PS, our typical recognizers (dark gray bars), and an additional 332 neurotypical observers from three independent studies for comparison (Faghel-Soubeyrand et al. 2019; Tardif et al. 2019; Fysh et al. 2020). **b)** Participants were engaged in a one-back task while their brain activity was recorded with high-density electroencephalography. The stimuli included objects from various categories, such as faces, objects, and scenes. Note that the face drawings shown here are anonymized representations used as substitutes for the actual face stimuli presented to our participants. **c)** Representational similarity analyses consisted in constructing brain representational dissimilarity matrices (RDMs) by comparing representational patterns (as characterized by EEG topographies) for all pairwise comparisons of stimuli, independently for each time-point and participants. Specifically, RDMs were constructed using cross-validated decoding performance between the EEG topographies at 4 ms intervals, providing a dynamic account of representational geometries unfolding after stimulus onset. **d)** To evaluate the temporal evolution of brain representations, a temporal generalization matrix was computed for each participant. This involved calculating all pairwise correlations between a participant’s time-resolved brain RDMs. A specific time-resolved brain RDM is considered to “generalize” to later time-resolved brain RDMs when it exhibits a positive correlation with them.

**Fig. 2:**
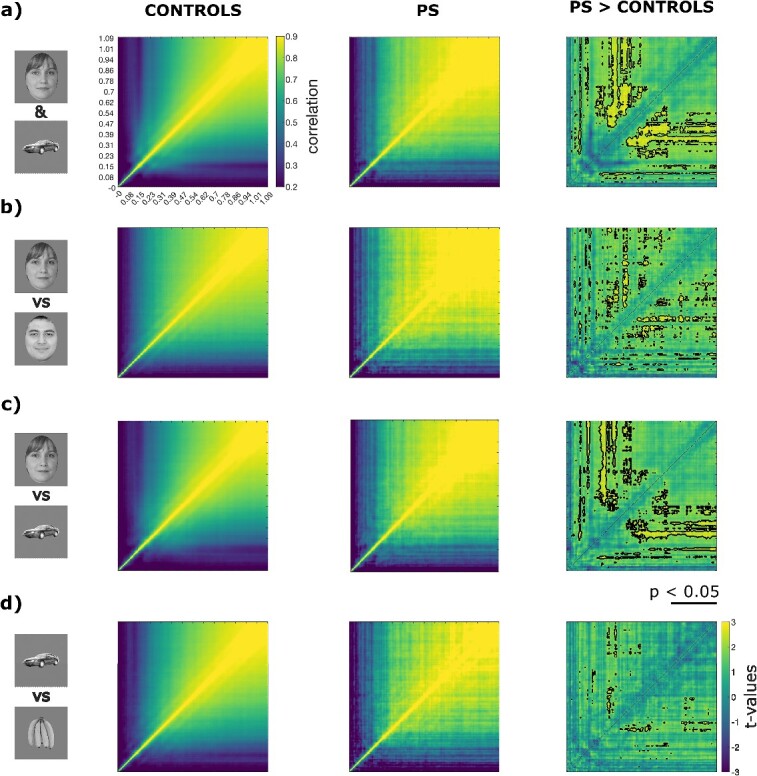
Temporal generalization of EEG representations across time in PS and controls. **a)** Temporal generalization over all pairwise stimulus comparisons. To assess the temporal evolution of brain representations, we computed a temporal generalization matrix for each participant. This process involved calculating pairwise correlations between time-resolved brain representational dissimilarity matrices (RDMs). The leftmost column displays the mean temporal generalization matrix of control participants. The yellowish square in the upper right section of the matrix indicates temporal generalization within the N170 time window. The central column illustrates the temporal generalization matrix of PS, which resembles that of the controls but is associated with earlier brain RDMs. This is most evident in the rightmost column, representing the difference between PS’s and controls’ temporal generalization matrices. Statistically significant regions in this contrast matrix are outlined in black (*P* < 0.05, uncorrected), with only positive differences reaching the threshold. **b)** Similar to a), temporal generalization matrices were computed, but this time specifically for a subset of time-resolved brain RDMs comparing pairs of face stimuli. **c)** Similar to a), temporal generalization matrices were computed, but this time specifically for a subset of time-resolved brain RDMs comparing pairs of face and nonface stimuli. **d)** Similar to a), temporal generalization matrices were computed, but this time specifically for a subset of time-resolved brain RDMs comparing pairs of nonface stimuli.

**Fig. 3:**
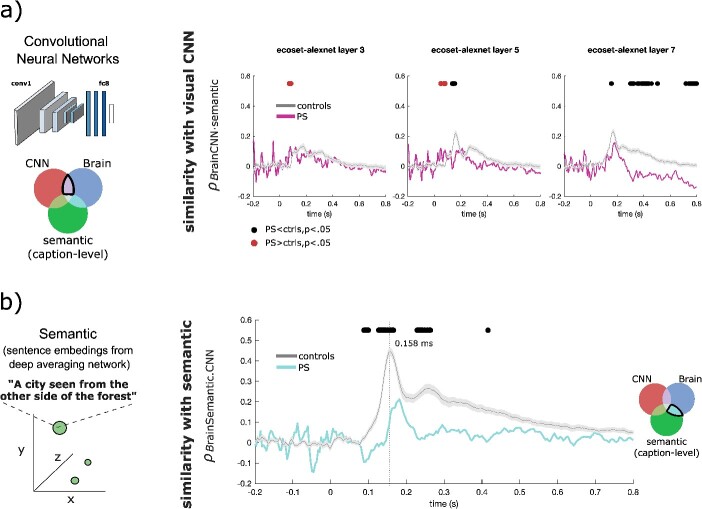
Comparison of brain representations with those of artificial neural networks of visual and semantic processing. **a**) Partial spearman correlation between brain RDMs and ecoset-trained AlexNet RDMs (removing shared correlation between brain and semantic model) is shown for PS (pink curve) and controls (gray curve). Each column shows different layer RDMs in ascending order from left to right. See Supplementary Fig. 2 for the same analysis on all the deep neural networks (DNN) layers. We found lower similarity of visual computations within the brain of PS compared to controls in layers 6 and 7 (black dots indicate significant contrasts in favor of controls, Howell-Crawford modified t-tests, *P* < 0.05; uncorrected), with differences peaking in higher-level DNN layer 7. We observed the opposite in layers 1 to 3 and, to a lesser extent in layer 5, at early time points (red dots indicate significant contrasts in favor of PS, Howell-Crawford modified t-tests, *P* < 0.05; uncorrected). Similar results were observed when comparing brains and DNN models without removing the shared information between brains and the semantic (caption-level) model (Supplementary Fig. 4). See Supplementary Fig. 3a and b for partial Spearman correlations for AlexNet trained on ImageNet and of VGGFace, respectively. **b**) Partial Spearman correlation with RDMs of the semantic model (excluding shared information between brain and AlexNet) was significantly lower in the brain of PS compared to controls (cyan curve; black dots indicate significant contrasts, *P* < 0.05; uncorrected). Similar results were observed when comparing brains and DNN models without removing the shared information between brains and the semantic (caption-level) model (see Supplementary Fig. 5). The shaded areas of all curves represent the standard error for the controls.

Patient PS recovered remarkably well from initially significant cognitive deficits with the support of medical treatment and neuropsychological rehabilitation. A couple of months after her injury she performed within the normal range at different non-visual tasks for which she was slightly impaired after the accident (e.g. calculation, short and long-term memory, visual imagery). She even resumed working as a kindergarten teacher only 2 years after her traumatic accident. Yet, her fine-grained visual discrimination abilities remained slower compared to controls, and she also presented reduced contrast sensitivity to high spatial frequency information (>22 cycles/degree) and a profound prosopagnosia with massively impaired face recognition abilities (Rossion et al. 2003). The patient complains of a severe difficulty at recognizing faces, including the ones of her kindergarten children, close relatives (husband, children, friends), as well as her own face. PS can correctly categorize (and draw) faces as a unique visual object and discriminate faces from other non-face objects or scenes, even when the images are briefly presented (Schiltz et al. 2006). She shows no difficulty at object recognition, even for subordinate-level discriminations (Rossion et al. 2003; Schiltz et al. 2006). Patient PS is perfect at all tests from the Birmingham Object Recognition Battery (BORB—(Riddoch and Humphreys 2022) showing preserved processing of low-level aspects of visual information (i.e. matching of basic elementary features), intact object matching from different viewpoints, and normal performance for object naming (Rossion et al. 2003; Table 1). Her reading abilities are also well preserved although slightly slowed down, her visual acuity (0.8 bilaterally) is within the normal range, and her visual field almost intact apart from a small left paracentral scotoma. As reported by Rossion et al. (2003), she is highly impaired on the Benton Face Matching Test (BFRT—(Benton and Van Allen 1972) scoring 27/54 (percentile 1). She performs also poorly on the Warrington Recognition Memory Test (WRMT—(Warrington and Shallice 1984), scoring 18/25 (percentile 3) a performance that characterizes her as impaired compared to controls. Over the years, patient PS developed strategies to infer a person’s identity by relying on external cues such as haircut, clothes, beard, glasses, gait, posture, or a person’s voice. Moreover, as revealed by the *Bubbles* response classification technique, patient PS uses suboptimal diagnostic information to recognize familiar faces, relying on the lower part of the face (i.e. the mouth region and external contours) instead of the most informative eye area (Caldara et al. 2005). A similar bias towards the mouth has been observed for the recognition of static facial expressions (Fiset et al. 2017) for which she is strongly impaired. Her ability to recognize the dynamic versions of the same facial expressions is nevertheless preserved (Richoz et al. 2015). Overall, PS is a very cooperative patient with extraordinarily preserved cognitive functions, sensory and motor skills and without any attentional deficits. She therefore represents an exemplary case to investigate the functional models of typical face processing.

### Behavioral tasks

#### Cambridge face memory test +

All participants were administered the CFMT long-form, or CFMT+ (Russell et al. 2009). In the CFMT+, participants are required to memorize a series of face identities, and to subsequently identify the newly learned faces among three faces. It includes a total of 102 trials of increasing difficulty. The duration of this test is about 15 min. EEG was not recorded while participants completed this test.

#### One-back task

The stimuli used in the main experiment consisted of 49 images of faces, animals (e.g. giraffe, monkey, puppy), plants, objects (e.g. car, computer monitor, flower, banana), and scenes (e.g. city landscape, kitchen, bedroom). The 24 faces (eight identities (four females) and three expressions: neutral, happy, and fearful) were taken from the Radboud Face dataset (Langner et al. 2010). For further details on stimulus processing steps, see (Faghel-Soubeyrand et al. 2024). In total, ~8 exemplars of each category (e.g. eight animals, eight objects, nine scenes, eight joyful/fearful/neutral faces) were presented to the participants during the EEG task*.*

These stimuli were presented during a one-back task where we measured high-density electroencephalographic (EEG) activity (Fig. 1b,c). Participants performed ~3200 trials in two recording sessions, which were separated by at least 1 day and by a maximum of 2 weeks. Participants were asked to press a computer keyboard key only on trials where the image was identical to the previous one (repetitions occurred with a 0.1 probability). They were asked to respond as quickly and accurately as possible. A trial unraveled as follows: a white fixation dot was presented on a gray background for 500 ms (with a jitter of ±50 ms); followed by a stimulus presented on a gray background for 600 ms; and, finally, by a white fixation dot on a gray background for 500 ms. Participants had a maximum of 1100 ms following stimulus onset to respond.

### Electroencephalography recording and preprocessing

High-density electroencephalographic data were continuously recorded at a sampling rate of 1024 Hz using a 128-channel BioSemi ActiveTwo headset (Biosemi B.V., Amsterdam, Netherlands). Electrodes’ impedance was kept below 20 μV. Data were collected at the University of Fribourg. Data were preprocessed using FieldTrip (Oostenveld et al. 2011) and in-house MATLAB code: continuous raw signal was first re-referenced relative to A1 (Cz), filtered with a band-pass filter [.01–80 Hz], segmented into trial epochs from −200 ms to 1100 ms relative to image onset, and down-sampled at 256 Hz. These EEG recordings were completed during the one-back task only.

### Representational similarity analysis

#### Brain representational dissimilarity matrices

For every participant, we trained a Fisher linear discriminant (5-fold cross-validation, five repetitions; Treder 2020) to distinguish pairs of stimuli from every 4 ms intervals of EEG response to these stimuli from −200 to 800 ms after stimulus onset (Cichy and Oliva 2020; Graumann et al. 2022). All 128 channels served as features in these classifiers. Cross-validated area under the curve (AUC) served as pairwise classification dissimilarity metric. By repeating this process for all possible pairs (1176 for our 49 stimuli), we obtained a representational dissimilarity matrix (RDM; Fig. 1c. see also Supplementary Fig. 1b).

#### Time generalization

We systematically characterized the differences in representational trajectories between PS and controls using a procedure similar to temporal generalization (King and Dehaene 2014). Specifically, we cross-correlated the RDMs across all time points after image onsets, creating a time × time temporal generalization matrix (TGM), indicating how similar is an RDM at a specific time window to other RDMs at other time windows, and compared this TGM between PS and controls using Howell-Crawford t-tests (see Fig. 1d, and Fig. 2).

#### Brain and deep neural networks comparisons

We compared our participants’ brain representations to those from visual and caption deep neural networks (DNN) using RSA (Charest et al. 2014; Kriegeskorte, Mur, and Bandettini, 2008; Kriegeskorte, Mur, Ruff, et al. 2008; Kriegeskorte and Kievit, 8/2013).

*Visual convolutional neural networks RDMs.* We used AlexNet (Krizhevsky et al. 2012) trained on ecoset, an ecologically valid image training dataset with faces, objects, etc. (Mehrer et al. 2021) one model of the visual computations along the ventral stream (Mehrer et al. 2021). We also used two other visual DNNs: AlexNet trained on ImageNet (Krizhevsky et al. 2012) and VGG-Face, a model specialized for faces (Parkhi et al. 2015). In all cases, our 49 stimuli were input to the DNN, and layer-wise RDMs were constructed comparing the unit activation patterns for each pair of images using Pearson correlations. These DNN process visual features of gradually higher complexity and abstraction along their layers (Güçlü and van Gerven 2015), from low-level (i.e. orientation, edges in shallow layers) to high-level features (e.g. objects and object parts in deeper layers).

*Caption-level semantic RDM.* We also used the caption-level semantic model derived by (Faghel-Soubeyrand et al. 2024). They asked five participants to provide a sentence caption describing each stimulus (e.g. “a city seen from the other side of the forest”), including those of faces (e.g. “a neutral female face”, “an unsure man’s face”), using the Meadows online platform (www.meadows-research.com). The sentence captions were fed as inputs in Google’s universal sentence encoder (GUSE; (Cer et al. 2018) resulting in a sentence embedding with 512 dimensions for each of our 49 stimuli. GUSE was trained to predict semantic textual similarity from human judgments, and its embeddings generalize to an array of other semantic judgment tasks (Cer et al. 2018). Faghel-Soubeyrand et al. (2024) then computed the dissimilarities (cosine distances) between the sentence embeddings across all pairs of captions, resulting in a caption-level semantic RDM for each of their participants. The GUSE embeddings forming the basis for the RDMs had good levels of agreement between the five captioners (r range across participants = 0.65–.71; mean r = 0.6823), and in turn, the resulting RDMs showed an average RDM spearman correlation between participants of r = 0.7116. Therefore, we used the average RDM for our analyses.

##### Representational dissimilarity matrices comparisons

We compared our participants’ brain RDMs to those from the vision (Fig. 3a, see also Supplementary Fig. 2) and caption-level semantic description (Fig. 3b) models described in the previous section using partial Spearman correlations (also see Supplementary Fig. 3 for comparisons with additional visual models). We accounted for the correlation between semantic and categorical models by partialling out the correlation with the last layer of AlexNet, and vice-versa. Specifically, for the brain-AlexNet correlations, each layer-brain RDM was correlated using partial Spearman correlation, partialling out the semantic RDM variance. For the brain-semantic model correlations, each semantic-brain RDM correlation was also done using partial Spearman correlation, partialling out the last layer of AlexNet RDM. Simple Spearman correlations (unconstrained on a third variable) were also computed and shown in supplementary materials (Supplementary Figs. 4 and 5).

### Group comparison and inferential statistics

All contrasts between PS and neurotypical controls were computed using Crawford-Howell modified t-tests for case-controls comparisons (Crawford and Howell 1998; Crawford and Garthwaite 2012). Non-parametric tests in single case studies have been demonstrated to be less reliable considering that there is no equivalent for non-parametric statistics that are reliable (Crawford et al. 2006). All time-resolved contrasts were computed from 0 to 800 ms after image-onset. To assess statistical significance in cases where we do not compare PS and controls we used nonparametric permutation tests (e.g. Nichols and Holmes 2002; Li et al. 2022).

## Results

### One-back task

Accuracies did not differ between age-matched and young controls subgroups either for face stimuli (t(16) = −0.3099, *P* = 0.761; t(16) = −0.9607, *P* = 0.3510) or non-face stimuli (t(16) = 1.2925, *P* = 0.215; t(16) = −.09704, *P* = 0.3463). Therefore, their data were aggregated into a single neurotypical control group.

#### Face-specific behavioral index

To assess the face-specific performance of PS and controls in a single individual score, we combined performance in the one-back task accuracies and response times of face and non-face trials using Principal Component Analysis (PCA). Specifically, face-specific performance in the one-back tasks was computed as a face vs. non-face performance contrast score ([face—non-face]/[face + non-face]) separately for accuracy and RTs, for each participant. We used PCA to extract projections explaining variance across these two variables (Calder et al. 2001; Calder and Young 2005). The first component, which explained 83.86% of the variance in performance across participants, is henceforth referred to as the face-specific performance score. PS significantly differed from neurotypical controls on this score (t(17) = −7.1571, *P* = 1.6053e-06; see Supplementary Fig. 1a), indicating typical face-specific behavioral deficits in this patient.

#### Cambridge face memory test long-form 

Within the control participants, CFMT+ scores did not differ between aged-matched and non–aged-matched control groups (t(16) = −0.8058, *P* = 0.4322). Their data were aggregated into a single control group. PS significantly differed from controls on this standard face identification ability score (t(17) = −2.7623, *P* = 0.0133).

#### Stability of neural code across time

A predominant assumption in cognitive neuroscience is that temporally early brain signal refers to low-level computations while later brain signal refers to higher-level computations (e.g. DiCarlo et al. 2012; Wiese et al. 2019). To investigate whether PS presents abnormal profiles of computations related to the lesioned cortical sites, we performed a variant of temporal generalization analyses (King and Dehaene 2014) using cross-correlated EEG RDMs across time. Specifically, correlation of EEG-RDMs across all time points after image onsets creates a (symmetric) time × time TGM. This symmetric temporal generalization matrix (TGM) indicates how representational geometries elicited at different time-points are similar (see Fig. 1d). We compared this TGM between PS and controls using Howell-Crawford t-tests (see Fig. 2). If the lesioned cortical sites perform critical computations on brain signals fed forward from early visual areas, we should observe excessive generalization of the representational geometries encoded in early time-points after stimulus onset for PS. Indeed, compared to controls, PS showed significantly higher correlation between her early representations (i.e. ~80-100 ms, i.e. around P100 (Luck et al. 1990) and late representations from around 230 ms to around 900 ms after image onset, as shown by the long trail of significant contrasts (Fig. 2a; black outline, *P* < 0.05; uncorrected). A similar and larger cluster was found between PS’s mid-latency representations around 300 ms, which generalized more than controls to late representations from ~400 ms until around 900 ms. Overall, thus, these results indicate that PS has more stable/less dynamic neural representations, with late EEG activity reflecting more similar representations to early EEG activity compared to neurotypical controls. We found essentially the same pattern of results when the TGM was computed on the face vs. face stimuli condition (Fig. 2b) and on face vs. non-face stimuli condition (Fig. 2c) but not on the non-face vs. non-face stimuli condition (Fig. 2d).

Altogether our temporal generalization results indicate that PS shows relatively less transformations in neural computations from early to late stages of her visual processing stream, especially for face stimuli (Cichy and Oliva 2020).

### Similarity with visual and semantic computational models

Our results suggest that the lesions in specific high-level cortical sites in PS contribute to an excessive temporal generalization of the face representations encoded in early time-points. Late stage processing must rely on inputs fed forward, and if those inputs come from early low-level processes, the high-level abstract representation relies on features that are weaker given the task at hand. But temporal generalization results do not explicitly reveal which kinds of computations may be impaired as a result of critical cortical sites being lesioned. To better understand the differences in computational trajectories between PS and controls, we compared brain RDMs to state-of-the-art computational models of vision and caption-level semantics. We assessed visual brain computations in PS and neurotypical controls by comparing their brain RDMs to those of the AlexNet-ecoset deep neural networks (DNNs).

The time courses of the partial Spearman correlation between brain RDMs and DNN layers 3, 5, and 7 (removing shared correlation between brain and semantic model) for PS and controls are shown in Fig. 3a (the partial Spearman correlations for all eight layers are shown in Supplementary Figs. 2 and 4, including individual control traces). Direct contrasts of PS’ correlation time courses with those of controls indicated reduced similarity with the RDMs of the visual DNN’s final layers (*P* < 0.05; uncorrected; layer 5: ~130–150 ms, layer 6: ~310–400 and 700–800 ms, layer 7: ~300–500 and 700–800 ms). These significant contrasts were present relatively late after image onset, and peaked at layer 7, which represent a higher proportion of high-level visual features (e.g. whole objects and object parts, (Güçlü and van Gerven 2015; Long et al. 2018). Interestingly, however, while similarity to these late visual representations were reduced in PS, similarity to earlier visual representations of the DNN (layers 1–5) were increased earlier on in this patient (see Supplementary Fig. 2, first two panels, *P* < 0.05; uncorrected; layer 1: 72-100 ms, layers 2–4: 72–84 ms, layer 5: 72–80 ms). This indicates that PS shows reduced similarity with high-level visual representations late after image onset and increased similarity with early visual representations early after image onset. We further tested the similarity to visual DNN representations of PS and controls by comparing them with similar convolutional networks trained either on faces-only (VGGface; (Parkhi et al. 2015); see Supplementary Fig. 3a) or objects-only (imagenet-trained AlexNet; (Krizhevsky et al. 2012); see Supplementary Fig. 3b). Overall this confirmed the impaired representational similarity to visual DNNs in the brain of PS in late stages of visual processing. However, the better alignment observed for PS early after image onset with early layers of ANNs was not observed in VGGface nor in AlexNet-imagenet. This discrepancy could potentially be explained by the images used in training these ANN models. VGGFace was trained with thousands of face identities in ~1,000,000 images. AlexNet-imagenet was trained with imagenet (Deng et al. 2009) which has been criticized for its lack of real world distribution of objects and categories (Beyer et al. 2020). The AlexNet-ecoset model was trained on a well-controlled diversified set of images aiming to improve the model’s ecological validity (Mehrer et al. 2021).

To reveal whether semantic computations (Barton et al. 2009; Schweinberger and Neumann 2016) could be affected in the brain of PS, we used a deep averaging network (Google Universal Sentence Encoder, GUSE (Cer et al. 2018)) to transform human-derived captions of our stimuli (e.g. “a city seen from the other side of the forest”) into semantic embeddings (points in a semantic space; for more details see Faghel-Soubeyrand et al. 2024). We computed partial Spearman correlations between the RDMs derived from this semantic model and the brain RDMs (excluding shared correlation between brain and the visual model) of PS and control participants. Direct contrasts revealed reduced partial Spearman correlations with these semantic computations in the brain of PS compared to controls (Fig. 3b, *P* < 0.05; uncorrected). This reduced similarity with semantic representations appeared as early as ~90 ms after image onset, with effects appearing until as late as around 400 ms. Note that similar results were observed when comparing brains and the semantic model without removing this shared information between brain and visual model (see Supplementary Figs. 4 and 5).

To better understand the features encoded in the different layers of the visual and semantic models, we compared the models with models constructed around the categorical features of our stimulus set. We constructed one-hot and multihot categorical models by encoding several dimensions as binary encoding vectors (including face gender, face emotion, face identity, animacy, object categories, etc.), and measuring the distance between these encoding vectors across all image pairs. The multihot RDM peaked in similarity with the semantic model. Correlation of categorical models with DNN RDMs, on the other hand, showed overall lower correlations with finer-grained categorical distinctions (e.g. the one-hot model encoding face gender information) and stronger dissociations between non-face stimuli along its latest layers. The correlation between the GUSE RDMs and relevant categorical models can be found in Supplementary Fig. 6. These correlations, as well as those with additional behavioral data on high-level human judgments (*n* = 32; see Supplementary Fig. 7), indicated that the semantic model contains rich information about animacy, face vs. non-face distinction, function and meaning of sentence description of images, as well as more fine-grained face-specific information about gender, emotion, and meaning at the level of sentence description. In other words, the semantic model encapsulates rich and diversified high-level conceptual information about images, including information about faces.

Overall, thus, contrasts with computational models showed that PS’s brain processing stream exhibits impairments peaking in higher-level visual (DNN layers 6–7) and semantic (caption-level) representations.

## Discussion

Finding brain correlates for the face individuation deficits seen in prosopagnosia has proven to be quite challenging, as highlighted in previous research (Anzellotti et al. 2014; Alonso Prieto et al. 2011; though there are exceptions, as seen in Liu-Shuang et al. 2016). In this study, we addressed this issue embracing a data-driven approach. We conducted high-density EEG recordings both on an individual with prosopagnosia (PS) and neurotypical controls while presenting various images from different categories. We employed RSA (Kriegeskorte, Mur, and Bandettini, 2008) to assess how the time-resolved representations of the visual stimuli relate to one another in the brain of PS and in the brains of controls, as well as how they relate to the representations of computational models. Our findings revealed that the temporal evolution of visual representations in PS’s brain follows an abnormal trajectory. Additionally, by comparing PS’s brain representations with those of computational models of vision and semantics, we gained insights into the nature of her computational deficits.

To investigate the changes in PS’s brain representations over time, we conducted a variant of temporal generalization analyses developed by King and Dehaene (2014). This involved correlating the time-resolved brain Representational Dissimilarity Matrices (RDMs) for an individual with all the RDMs of that same individual. In the case of neurotypical participants, we observed the expected temporal generalization of the brain RDMs within the N170 temporal window. This indicates that the brain’s visual representation at this point in time is relatively stable as time progresses. In other words, additional computations after 170 ms do not significantly alter the brain representations of control participants. However, in the case of PS, we noted atypical generalization patterns. Specifically, the brain representations around the P100 time window were abnormally similar to later representations, suggesting an excessive generalization of early visual representations. This overgeneralization was primarily associated with the similarity between brain activity in trials involving a face and a nonface stimulus and, to a lesser extent, between two faces, but not between two non-face stimuli. This implies that the key differences lie in how the brain represents faces between 80 and 100 ms in PS, possibly due to critical computations in cortical sites that affect the feedforward signals from early visual areas responsible in the typical brain for achieving a stable N170 face representation.

To investigate this possibility, we conducted a comparative analysis of the RDMs derived from PS’s brain with those generated by a deep neural network (DNN) trained to distinguish objects and faces. We assumed that this DNN model represents a nearly optimal sequence of visual stimuli representations for object recognition. Our findings revealed that the neural computations underlying PS’s brain activity in the early layers exhibited a closer alignment with the model of vision than those of neurotypical individuals. This suggests that PS’s early brain representations are, indeed, better building blocks than those of control participants for recognizing faces and nonface visual stimuli. As we delved into the middle layers of the DNN, both PS and neurotypicals showed a relatively similar alignment, and in the later layers, the brain representations in neurotypical individuals became more akin to those of the model, that is, more efficient.

It is worth noting that late layers in visual DNNs have previously been associated with processing in the human infero-temporal cortex (hIT; Güçlü and van Gerven 2015; Khaligh-Razavi and Kriegeskorte 2014; Jiahui et al. 2023), with a peak in the FFA (Khaligh-Razavi and Kriegeskorte 2014). These layers are functionally connected to higher-level visual feature representations, encompassing various aspects of objects, including parts, whole objects, and viewpoint-invariant features. These findings are thus in agreement with the previously documented impairments in both whole face (Ramon et al. 2016) and feature representations (Caldara et al. 2005; Ramon et al. 2016; Fiset et al. 2017) in patient PS. One interesting observation from the model to brain comparisons performed here is that the different model layers show similar onsets of peak correlations. This is an observation that has previously been documented (Cichy et al. 2016). Neural traces, as assessed through EEG, can exhibit diverse components of feedforward and feedback signals. In contrast, deep convolutional neural networks, such as those employed in our study, are intentionally designed to be hierarchical, featuring progressively abstracted information from pixel inputs as we move deeper into the network layers. Therefore, the absence of a strictly monotonic relationship between peak onset and model layer is to be expected.

Associations between brain activity and higher-level semantic computations have only recently gained attention in the field (Doerig et al. 2022; Dwivedi et al. 2021; Faghel-Soubeyrand et al. 2024; Popham et al. 2021). Here, we demonstrated that the representational geometry of PS exhibits a substantial, albeit significantly reduced, correlation with those of a model of caption-level semantics (Cer et al. 2018; Doerig et al. 2022; Frisby et al. 2023; Faghel-Soubeyrand et al. 2024) when compared to controls. Further analyses suggest that this semantic model encapsulates a wealth of information about various aspects of face categories, including gender and emotion, differentiation between faces and non-face stimuli, as well as the functional aspects of objects, faces, and scenes. Our findings unequivocally illustrate a strong connection between these semantic brain computations and significant alterations in the ability to recognize faces, underscoring the importance of these computations in the context of face recognition (Bruce and Young, 1986; Duchaine and Yovel 2015; Faghel-Soubeyrand et al. 2024).

In conjunction with similar computational characterizations of brain representations in individuals with “super-recognition” of faces (Faghel-Soubeyrand et al. 2024), our findings suggest a gradient of neural computations spanning from the lower end to the higher end of face-recognition abilities. For instance, in the study by Faghel-Soubeyrand et al. (2024), super-recognizers exhibited increased similarity with mid-level visual and semantic computations around the N170 and P600 time windows, respectively. In contrast, our results demonstrate that PS shows reduced similarity with visual and semantic computations. Specifically, PS’s neural computations, especially in the realm of semantic processing, appear to be affected at a much earlier stage and to a greater extent than those of super-recognizers. While semantic brain computations were enhanced around the P600 time window in super-recognizers, PS exhibited reduced similarity as early as the P100, persisting throughout the N170 and N400 time windows. These observations indicate that PS’s deficits in neural computations commence relatively early along the conventional processing pathway.

To the best of our knowledge, this study presents the first analysis of the fine-grained temporal progression of brain representations in prosopagnosia, alongside a state-of-the-art computational characterization of these representations. This comprehension of impaired perceptual representations not only paves the way for novel approaches to patient rehabilitation but also holds promise in uncovering and potentially diagnosing subtle deficits in perception and cognition across diverse clinical populations. These advancements have been facilitated by recent technological progress, which has significantly contributed to the findings of this study.

## Supplementary Material

Supplementary_Materials_CerCor_bhae211

## Data Availability

Data are available from the corresponding authors upon request.

## Code availability

The MATLAB and Python codes used in this study will be available upon request.
