# Supplementary material for: Neural computations in prosopagnosia
Source: Cereb Cortex. 2024 May 24;34(5):bhae211. doi: 10.1093/cercor/bhae211 (PMC11127037; doi:10.1093/cercor/bhae211)
Supplement: Supplementary_Materials_CerCor_bhae211 [file supplementary_materials_cercor_bhae211.pdf]

Supplementary Materials.

Reliability of neural dynamics measured with RSA

Measuring brain dynamics of brain-lesioned patients can be arduous using raw electrophysiological topographies (Alonso Prieto et al., 2011). We computed the reliability of brain representations in a similar way as in (Charest et al. 2014). We assessed whether we could measure reliable brain representations of brain-lesioned PS by computing inter-session reliability of brain Representational Dissimilarity Matrices (RDMs). We computed the Spearman correlation between EEG RDMs computed from recording day 1 and recording day 2 at every 4 ms steps. Significance was assessed using permutation testing. Specifically, we created, for each participant and at each 4 ms step, a null distribution of 1000 brain-to-brain correlations using an RDM in which rows and columns indices were randomly shuffled. The timecourse of these correlations, shown in **Supplementary Figure 1b**, indicates significant (*ps*<.05, permutation testing) reliability of brain representations of both neurotypicals and PS across most time points after image onset, peaking in the N170 window (160 ms and 180 ms for controls and PS, respectively; r_{peak_ctrls} = .4316; r_{peak_PS} = .2638). PS showed surprisingly high SNR across sessions, her correlation time course being on par with or even higher than the neurotypical participants' reliability scores around 80 ms and between 500 and 800 ms (see Supplementary Figure 1B; blue dots).

Comparison between deep neural network models of vision and classical category-based models

The Spearman correlation between the CNN (AlexNet trained on ecoset) RDMs and relevant categorical models can be found in Supplementary material (see **Supplementary Figure 6**). Here, all comparisons between brain and CNN were made at the level of the complete RDM matrices (including face and non-face stimuli; see method section). This was done considering that the brains of two individuals might differ on *how* they resolve the same task, even in presence of weak or presumably absent differences in decoding (e.g. no impairment for non-face objects exemplars decoding in the brain of PS, as we show in figure 3). That is to say that the underlying neural code in PS might be different than that of typical participants for face and non-face objects. Furthermore, we inspected brain-CNN correlations only for latencies showing reliable representations across days according to RDM reliability traces (that is, until 800 ms; see figure 2).

Supplementary Figures

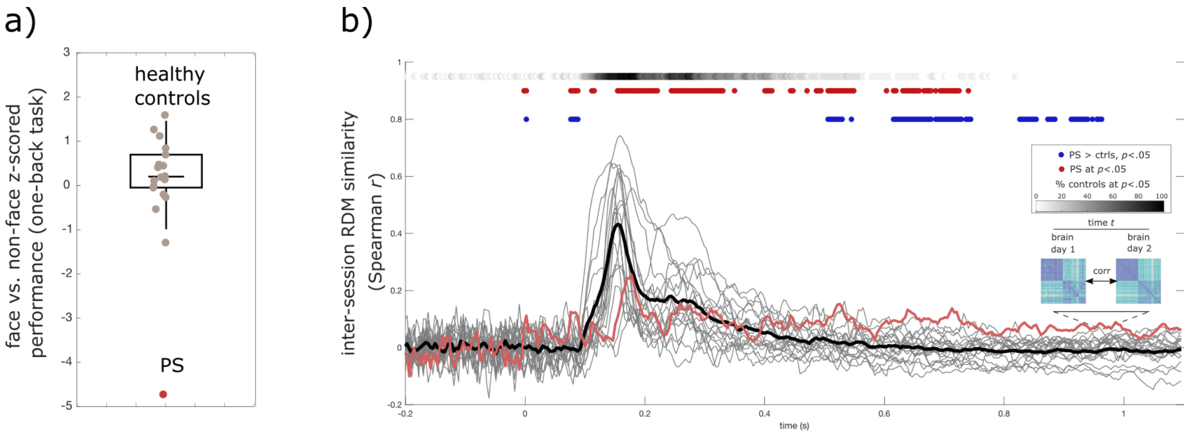

Supplementary Figure 1. Behavioural performance and reliability of brain representations across time. (a) Individual face-specific one-back task accuracies transformed into z-scores. The performance of PS was significantly inferior to the performance of controls (*t(17)* = -7.1571, *p* =1.6053e-06). **(b)** Brain Representational Dissimilarity Matrices (RDMs) computed on different recording days were cross-correlated within participants for patient PS (red line) and neurotypical participants (grey lines indicate individuals participants, black line indicate control-averaged). PS brain RDMs showed significant inter-session reliability across most time windows (*p*<.05, permutations; uncorrected). For comparison, the percentage of control participants with significant cross-session correlations is shown on top for every time point, with darker points indicating higher percentage of neurotypicals with reliable brain RDMs. Overall, PS had reliability coefficients similar to those of controls.

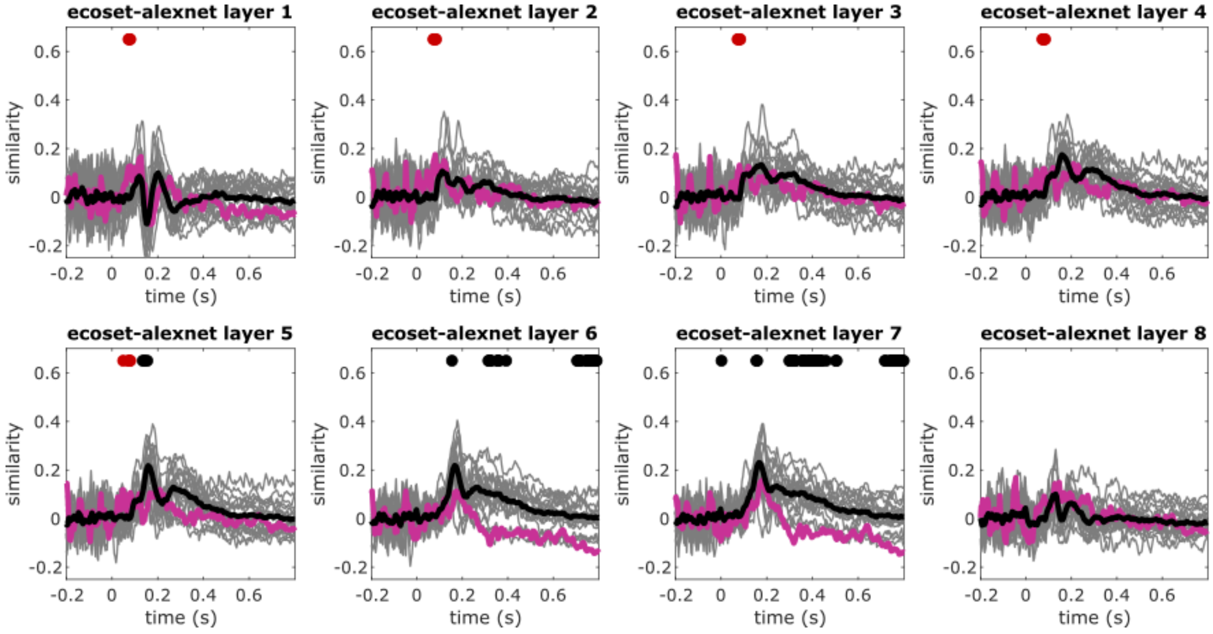

Supplementary Figure 2. Individual time course of similarity with ecoset-trained AlexNet. Partial Spearman correlation between brain RDMs and ecoset-trained AlexNet RDMs (removing shared correlation between brain and semantic model) is shown for PS (pink curve) and all control participants (grey curves; black curve shows the mean across controls). Each panel shows different layer RDMs in ascending order from left to right. In early layers, we found significantly larger similarity of visual computations within the brain of PS compared to controls (red dots indicate significant contrasts; Howell-Crawford modified t-tests, $p<.05$; uncorrected). In contrast, for the mid and late layers, we found overall lower similarity of visual computations within the brain of PS compared to controls (black dots indicate significant contrasts, Howell-Crawford modified t-tests, $p<.05$; uncorrected), with differences peaking in higher-level CNN layer 7.

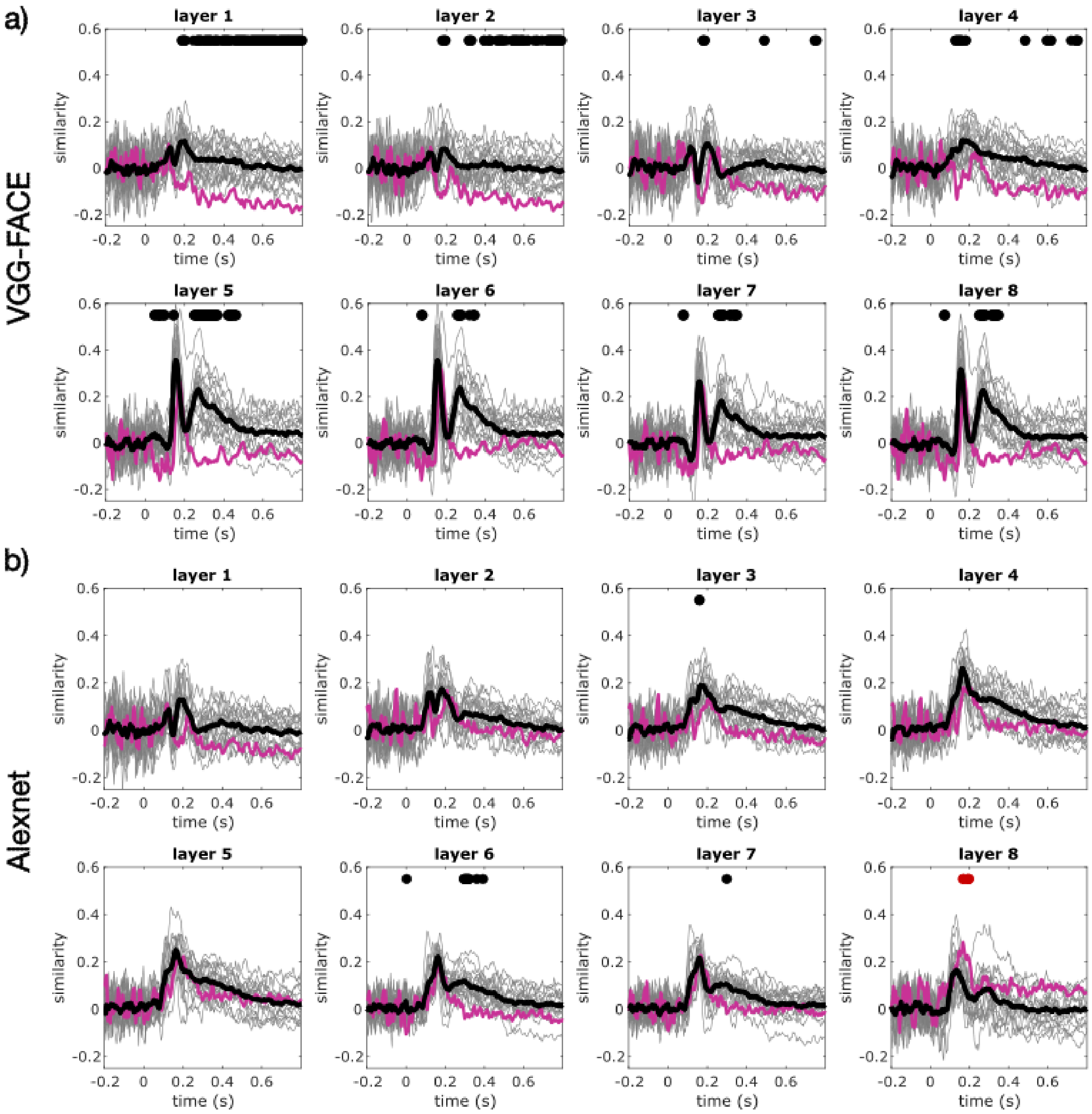

Supplementary figure 3. Time course of similarity with imagenet-trained AlexNet and VGGface model. a) Time course of (Spearman) partial correlation between brain RDMs and imagenet-trained AlexNet RDMs (constrained on the semantic model) is shown for PS (pink curve) and controls (grey curve). Each plot shows correlations with a different layer RDM. **b)** Time course of (Spearman) partial correlation between brain RDMs and face-trained VGGface RDMs (constrained on the semantic model) is shown for PS (pink curve) and controls (grey curve). Overall, we found lower similarity of visual computations within the brain of PS compared to controls (black dots indicates significant contrasts PS< controls, Howell-Crawford t-tests, *p*<.05), indicating similar results to those observed with the ecoset-trained AlexNet, in **Figure 3** and **Supplementary Figure 2**).

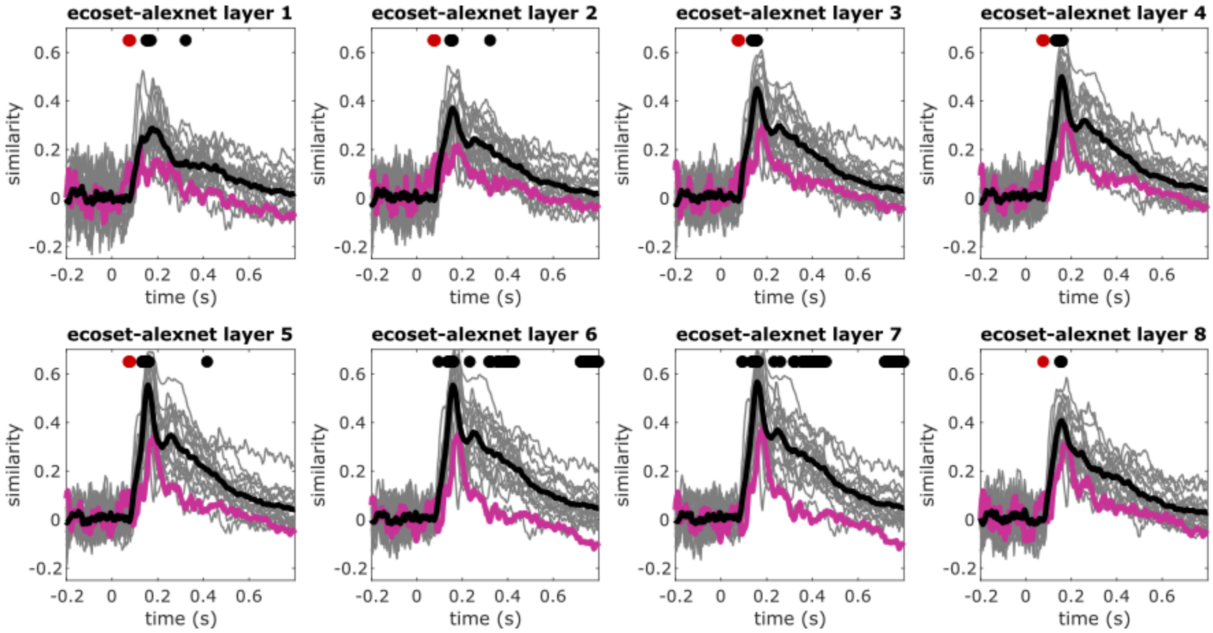

Supplementary Figure 4. Time course of ecoset-derived visual computations, unconstrained. **a**) Time course of correlation between brain RDMs and ecoset-trained AlexNet RDMs is shown for PS (pink curve) and controls (grey curves; black curve shows the mean across controls). Each plot shows correlations with the RDMs from a different layer. In early layers, we found significantly larger similarity of visual computations within the brain of PS compared to controls in early time points (red dots indicate significant contrasts PS > controls; Howell-Crawford modified t-tests, p<.05; uncorrected), and the opposite for later time points (black dots indicate significant contrasts PS < controls, Howell-Crawford t-tests, p<.05). In mid to late layers, we found overall lower similarity of visual computations within the brain of PS compared to controls (black dots indicate significant contrasts PS < controls, Howell-Crawford t-tests, p<.05). These results are similar to the results of the constrained analysis shown in **Figure 3** and **Supplementary Figure 2**.

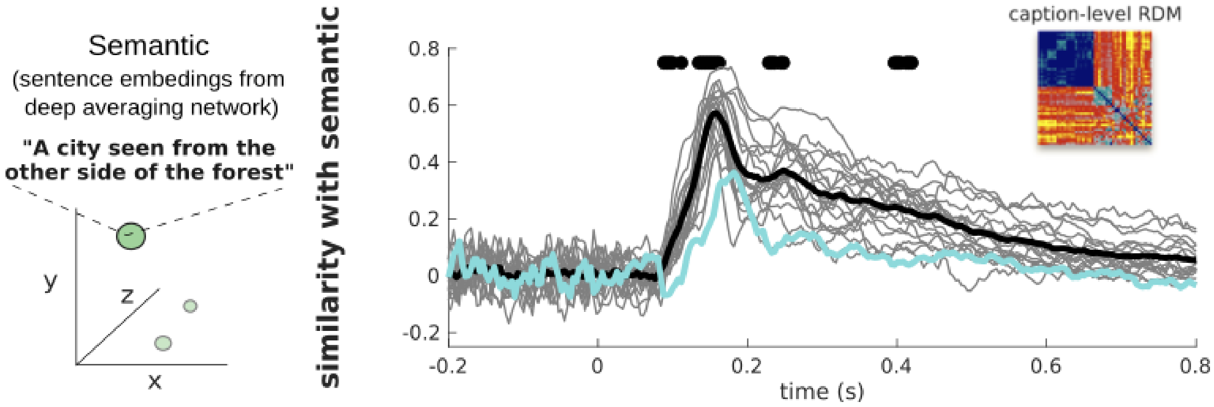

Supplementary figure 5. Time course of semantic computations, unconstrained. a) Time course of correlation between brain RDMs and semantic model RDM is shown for PS (pink curve) and controls (grey curves; black curve shows mean across controls). Overall, we found lower similarity of semantic computations within the brain of PS compared than those of controls (black dots indicate significant contrasts, Howell-Crawford t-tests, $p<.05$), which is similar to the results of the constrained analysis shown in **Figure 3**.

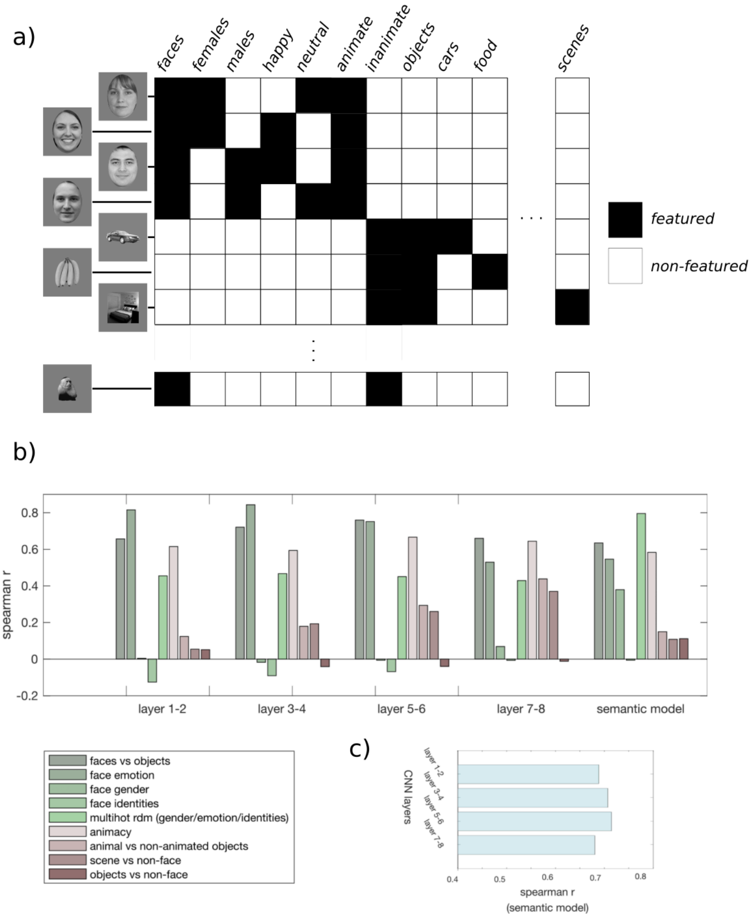

Supplementary figure 6. Comparison of Artificial Neural Network models to categorical models. Panel (a) shows the encoding model strategy used to create categorical models. In this cartoon example, each row represents a stimulus, and each column represents a dimension. Black cells in the table show dimensions that feature in the stimulus, and white cells, dimensions that don't feature in the stimulus. One-hot encoding RDMs are constructed by taking a single column (e.g. faces) of this encoding matrix and computing the distance between all pairs of items. Multi-hot encoding RDMs are computed by taking multiple columns of the encoding matrix and computing the distance across all pairs of stimuli. Panel **(b)** shows the correlation (Spearman) between the model RDMs and relevant categorical models from our stimulus set (shades of green representing different face information). Spearman Correlations with CNN layer RDMs (CNN layer 1-2, 3-4, 5-6, 7-8) are shown from left to right, with the semantic model on the far right. Overall, this showed that the semantic model contains categorical information about face vs. objects, animacy, and more fine-grained categorical information about face-gender/face-emotion. A model integrating face-gender, emotion and identity (multi-hot RDM) peaked in similarity with the semantic model. Correlation of categorical models with CNN RDMs, on the other hand, showed overall lower relationship with finer-grained face-gender categories and stronger dissociation between non-faces objects (scenes vs. non-faces, objects vs. non faces) along its deeper layers. Panel **(c)** shows the (spearman) correlation between ecoset-trained AlexNet and the GUSE semantic model across the CNN layers.

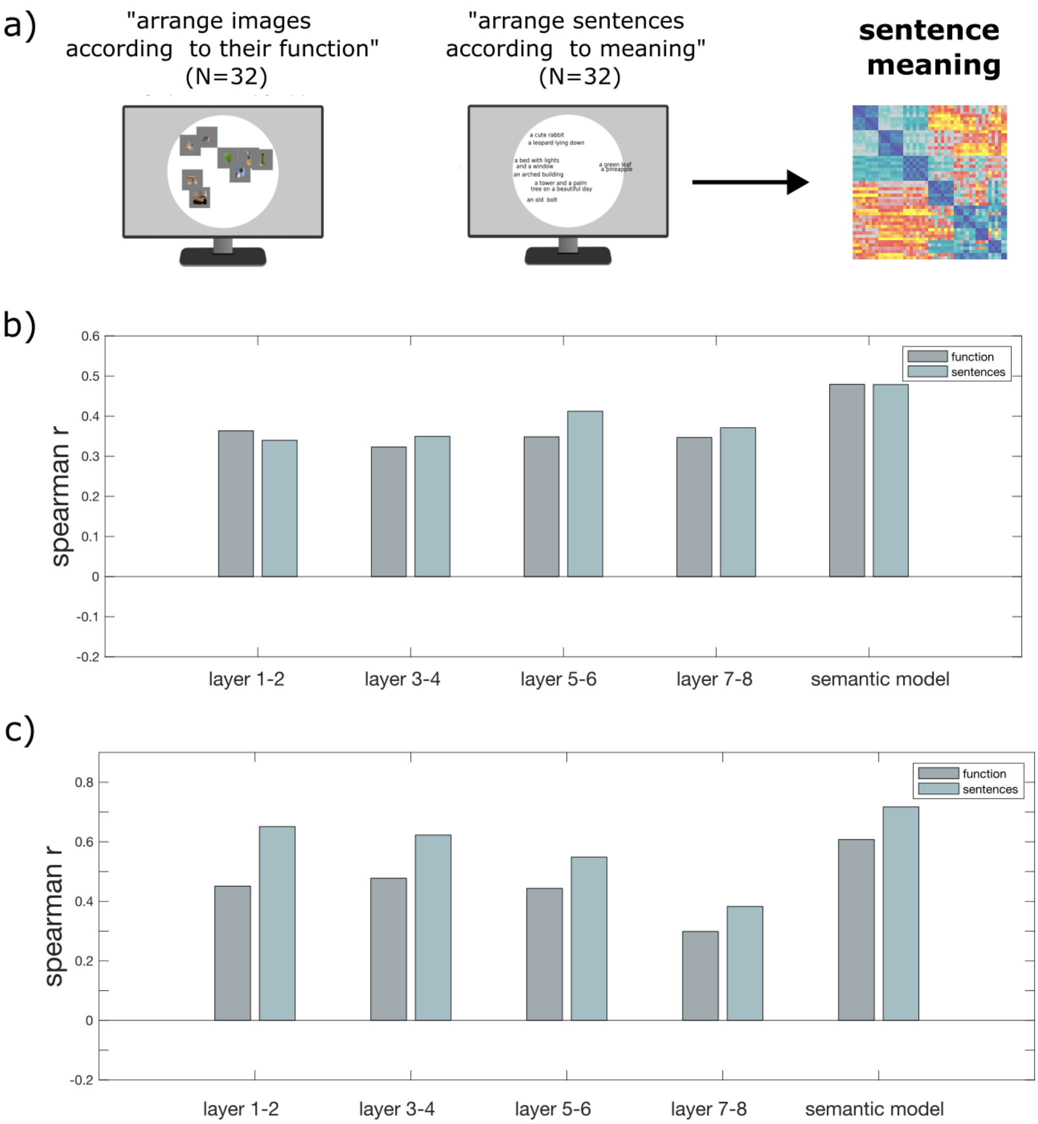

Supplementary figure 7. Comparison of Artificial Neural Networks models to human tasks representations. a) RDMs were computed from different *human behavioural* judgements of additional participants from an online study (N=32) aimed to capture judgements of function and sentences meaning. In the function judgement task, participants were asked to evaluate the function similarities of the 49 object/face/scene images used in the main experiment; in the sentence task, they were instructed to judge the meaning similarities of the 49 sentence captions describing these images. Participants arranged the images/sentences on a computer screen inside a white circular arena according to the task instructions using simple drag and drop operations. **b)** The first panel shows the partial correlation (Spearman) between the CNN model RDMs and relevant behavioural models from all our stimuli set, regressing CNN RDM for the semantic model, and vice versa (as shown in main figure 4). This revealed overall a peak association of human judgements about function and sentence meaning about our stimuli with the semantic model. **c)** The second panel shows identical analyses, but focused on face stimuli. It shows a clear prominence of the higher-level models, peaking in the semantic model, to capture high-level face information about human judgements of function and meaning.
